# A266 TISSUE-SPECIFIC FUNCTION OF THE CIRCADIAN GENE, *BMAL1*, DURING COLITIS RECOVERY

**DOI:** 10.1093/jcag/gwad061.266

**Published:** 2024-02-14

**Authors:** Z Taleb, V Carmona-ALcocer, R Wan, P Karpowicz

**Affiliations:** Biomedical Science, University of Windsor Faculty of Science, Windsor, ON, Canada; Biomedical Science, University of Windsor Faculty of Science, Windsor, ON, Canada; Biomedical Science, University of Windsor Faculty of Science, Windsor, ON, Canada; Biomedical Science, University of Windsor Faculty of Science, Windsor, ON, Canada

## Abstract

**Background:**

The circadian clock is a self-sustained molecular oscillator which drives 24-hour physiological rhythms in nearly every cell of the body. It consists of the genes *Bmal1* and *Clock* that positively regulate *Cry* and *Per*, their negative regulators, resulting in a 24-hour transcription/translation feedback cycle. Shift work, which causes disruptions to 24-hour physiological rhythms, has been linked with an increased incidence of inflammatory bowel disease (IBD).

**Aims:**

We have previously established that mice lacking the non-redundant circadian regulator, *Bmal1*, exhibit more severe colitis compared to controls. The loss of *Bmal1* also negatively impacts post-colitis regeneration and recovery. However, the role of *Bmal1* in specific cell types remains unknown in regard to regenerative capacity following colitis. The intestinal stem cell niche includes stromal cells that provide signaling factors to regulate the proliferation of epithelial cells in the colonic crypt.

**Methods:**

To test the role of the circadian clock in these cell types, we conditionally disrupted *Bmal1* in intestinal epithelial versus stromal cells using *Vil*^*Cre/+*^*;Bmal1*^*flox/flox*^ and *Pdgfra*^*Cre/+*^*;Bmal1*^*flox/flox*^ mice, respectively. Dextran Sulfate Sodium (DSS) was applied to induce acute colitis, disease progression was evaluated, and regeneration at different stages of recovery was analyzed. We hypothesize that the cell-specific disruption of *Bmal1* in both intestinal epithelial and stromal cells negatively impacts the regenerative capacity following colitis, leading to delayed recovery and increased inflammation.

**Results:**

No differences in survival and colitis disease activity were observed between *Vil*^*+/+*^*;Bmal1*^*flox/flox*^ control and *Vil*^*Cre/+*^*;Bmal1*^*flox/flox*^ but increases in lesion number, inflammation, and disrupted recovery are evident in *Vil*^*Cre/+*^*;Bmal1*^*flox/flox*^ colons histologically. Whereas control animals are able to recover two weeks following DSS treatment, regenerative proliferation is still present in conditional knockouts. Thus, *Bmal1* is required in intestinal epithelial cells for efficient repair. Preliminary results in the *Pdgfra*^*Cre/+*^*;Bmal1*^*flox/flox*^ conditional stromal mutanats do not show differences in survival or disease severity, and we are currently evaluating effects on regeneration.

**Conclusions:**

Our results support a critical role for *Bmal1* in intestinal epithelial cells during post-colitis regeneration and recovery.

**Funding Agencies:**

CCC, CIHR

